# Are perceived stress, depressive symptoms and religiosity associated with alcohol consumption? A survey of freshmen university students across five European countries

**DOI:** 10.1186/1747-597X-7-21

**Published:** 2012-05-28

**Authors:** Rene Sebena, Walid El Ansari, Christiane Stock, Olga Orosova, Rafael T Mikolajczyk

**Affiliations:** 1 Department of Psychology, Faculty of Arts, PJ Safarik University, Kosice, Slovak Republic; 2 Department of Sport and Exercise, Faculty of Applied Sciences, University of Gloucestershire, Gloucester, UK; 3 University of Southern Denmark, Unit for Health Promotion Research, Esbjerg, Denmark; 4 Department of Educational Psychology and Health Psychology, Faculty of Arts, PJ Safarik University, Kosice, Slovak Republic; 5 Department of Clinical Epidemiology, Bremen Institute for Prevention Research and Social Medicine, Bremen, Germany; 6 Bremen Insitute for Prevention Research and Social Medicine, Achterstr. 30, D-28359, Bremen, Germany

**Keywords:** Perceived stress, Depressive symptoms, Problem drinking, Drinking frequency, Religious faith, Freshmen university students

## Abstract

**Background:**

The aim of this study was to investigate the association of perceived stress, depressive symptoms and religiosity with frequent alcohol consumption and problem drinking among freshmen university students from five European countries.

**Methods:**

2529 university freshmen (mean age 20.37, 64.9% females) from Germany (n = 654), Poland (n = 561), Bulgaria (n = 688), the UK (n = 311) and Slovakia (n = 315) completed a questionnaire containing the modified Beck Depression Inventory for measuring depressive symptoms, the Cohen’s perceived stress scale for measuring perceived stress, the CAGE-questionnaire for measuring problem drinking and questions concerning frequency of alcohol use and the personal importance of religious faith.

**Results:**

Neither perceived stress nor depressive symptoms were associated with a high frequency of drinking (several times per week), but were associated with problem drinking. Religiosity (personal importance of faith) was associated with a lower risk for both alcohol-related variables among females. There were also country differences in the relationship between perceived stress and problem drinking.

**Conclusion:**

The association between perceived stress and depressive symptoms on the one side and problem drinking on the other demonstrates the importance of intervention programs to improve the coping with stress.

## Background

Alcohol use during early adulthood presents a serious public health problem in many countries [1,2]. Studies have shown that heavy drinking and related problems are prevalent among young people, regardless of whether they attend college or not [3]. The majority of studies have found higher rates of alcohol-related problems in students compared to non-students [4-6]. In contrast, a recent study showed that college students drink less frequently than their non-college peers, but when students do drink, they tend to drink in greater quantities than non-students [7]. Among university students, first year freshmen display particularly high levels of alcohol consumption [8]. On the one hand, first year at university is a developmental transition to new responsibilities in absence of well-established networks of social support. On the other hand, it also represents freedom, liberty and fewer restrictions due to living away from parents [9]. Both aspects can increase the use of alcohol among students.

Generally, alcohol consumption has been linked to mental health problems e.g. perceived stress or depressive symptoms [10-15]. Depressive symptoms (based on DSM-IV) include sadness, anxiety or empty feelings; decreased energy; loss of interest in usual activities; sleep disturbances; weight gain/loss; feelings of worthlessness; suicidal thoughts; difficulty in concentrating or making decisions. Tension reduction theory contends that tension-producing circumstances (i.e. stressors) could lead to increased drinking [16-18]. As alcohol is perceived to reduce tension high levels of stress are therefore associated with drinking [19,20], and depressive symptoms might also result in increased alcohol consumption [21]. Indeed, college students consume alcohol to: potentially relax or relieve tension; celebrate; feel comfortable with the opposite gender; as a reward for working hard; and to get away from troubles [22].

Religiosity is involved in the coping processes against stress and depressive symptoms [23,24]. Religious coping entails dealing with stress, which involves a religious perspective. Such religious coping includes prayer, congregational support, pastoral care, and religious faith [25]. An alternative value system, which could be part of religious coping, may also improve the coping mechanism with respect to alcohol drinking behavior. The ‘net’ effects of the academic, social or economic stressors on student wellbeing might well depend on the extent of availability of coping resources in order to ‘buffer and balance’ the stress. Certainly, studies have demonstrated an inverse relationship between religiosity and alcohol use. For instance, students who reported that “religion is important in my life” had a lower frequency of heavy drinking in Nova Scotia, Canada [26] and in the USA [27,28]. Similarly, American students who used religious coping tended to drink less alcohol [29]. Such evidence suggested that religiosity could be a potential confounder or even factor that modifies the association between mental health problems and alcohol consumption.

University students in various Eastern and Western European countries differ in terms of the reported levels of perceived stress and depressive symptoms [30], alcohol consumption frequency, and the proportions of students with problematic alcohol use [1,31]. Based on past history, there are still differences between countries of Eastern and Western Europe in terms of social life, health knowledge/literacy, individual habits, sense of power/powerlessness over health, and health-related lifestyles [32]. Such lifestyle factors have been suggested to contribute to differences in health and well-being across Western and Eastern Europe [33]. Further, given the different religious traditions with majority status in each country, personal religiosity has a potentially different association with alcohol consumption across the countries.

Although the problem of alcohol use among university students is evident almost globally [34-38], comparative studies using the same alcohol measures, identical mental health indicators and similar methodologies remain limited across countries. The current study bridges this gap by employing the same measures and identical indicators of alcohol consumption, depressive symptoms, perceived stress, and religiosity across five Eastern and Western European countries. For three of the five countries (Germany, Bulgaria and Poland), descriptive results of alcohol consumption [31] and of depressive symptoms and perceived stress [39] have been published recently. However, the associations between alcohol consumption and depressive symptoms and perceived stress were not assessed.

### Aim of the study

The aim of the current study was to assess the associations of perceived stress and depressive symptoms with frequency of alcohol use and problem drinking among university students from five European countries, as well as to examine the role of religiosity for these associations. The specific objectives of the study were to:

· Assess whether perceived stress is associated with a high alcohol use frequency and problem drinking and whether this association differs by country and/or by sex. It was hypothesized that a higher level of perceived stress would be associated with more frequent alcohol use and problem drinking.

· Assess whether depressive symptoms are associated with high alcohol use frequency and problem drinking. It was hypothesized that a higher level of depressive symptoms will be associated with more frequent alcohol use and problem drinking.

· For both the above objectives, the role of religiosity was examined. It was hypothesized that religiosity modifies the associations between perceived stress or depressive symptoms on the one hand, and the frequency of alcohol use and problem drinking on the other.

## Methods

### Sample

The analysis described in this paper is based on data collected among university students in five countries from Western and Eastern Europe as part of the Cross-National Student Health Study (CNSHS) [40]. The participating universities were selected on basis of personal contacts of the researchers. These included: Bielefeld University in Bielefeld, Germany; the Catholic University of Lublin, Lublin, Poland; the University of Sofia, Sofia, Bulgaria; the University of Gloucestershire, United Kingdom; and, three universities in Kosice, Slovakia – PJ. Safarik University, the University of Veterinary Medicine and the Technical University. The campuses of all participating universities are situated in urban environments and alcohol consumption was not restricted on the campuses. The survey was conducted in Germany, Bulgaria and Poland (May 2005), in the UK (May 2007) and in Slovakia (May 2008).

A self-administered questionnaire was distributed to first-year students during regular classes of courses which were selected in order to obtain about one third of the sample each from the natural sciences, the social sciences, and business and law. In the UK and Slovakia, students cross all years were sampled, but only first year students were included in the current analysis. The overall response rate was 74% and the final sample included 2529 students. In order to increase the accuracy of self-reports, students were assured that their answers would remain confidential, and data protection was observed at all times.

### Informed consent and ethics

Participation in the study was voluntary and anonymous. Students were informed that by completing the questionnaire they were providing their informed consent to participate. They were also informed that they could terminate the participation at any point while filling out the questionnaire. The permission to conduct the study was granted by the participating institutions: Bielefeld University in Bielefeld, the Catholic University of Lublin, Lublin, the University of Sofia, the University of Gloucestershire, the University of PJ Safarik, the University of Veterinary Medicine and Pharmacy in Kosice and the Technical University of Kosice.

### Measures

The questionnaire was developed in German and then translated into Polish, Bulgarian, English and Slovak using two independent forward translations for each language. The research team reviewed any cases of disagreement and the authors familiar with the respective languages (usually native speakers) made final decisions. The translations focused on and ensured the equivalence of meaning [41], and the assessment of the same attributes/features in each cultural group/country [42], as cross-cultural and cross-language equivalence require attention [43].

#### Frequency of alcohol consumption

Frequency of alcohol consumption was measured using the question: “Over the past three months how often have you drunk alcohol, for example, beer?” The response options were: “never,” “once a week or less,” “once a week,” “a few times each week,” “every day,” “a few times each day”. Abstainers were excluded from the analyses. For the purpose of analysis, this variable was dichotomised into “Low” (drinking once a week or less) versus “High” (drinking several times per week).

#### Problem drinking

In order to gather data on problem drinking we included an alcoholism-screening test, the CAGE test [44]. CAGE is a brief screening instrument comprising four short questions: “Have you ever felt you should cut down on your drinking?”; “Have people annoyed you by criticizing your drinking?”; “Have you ever felt bad or guilty about your drinking?”; and, “Have you ever had a drink in the morning to get rid of a hangover?” Each question is answered with either “yes” or “no.” Two or three affirmative answers suggest problem drinking, while four positive responses indicate serious suspicion of alcohol dependence [44]. Respondents were classified as non-problem drinkers (<2 positive answers) and problem drinkers (≥2 positive answers).

#### Demographic and economic variables

Students’ sex and age were based on individuals’ self-reports in the survey. Respondents’ subjective financial situation was measured by a single item: “How sufficient is your income?”, with a 4-point response scale ranging from 1 = ‘totally sufficient’ to 4 = ‘not sufficient at all’). To assess religion, the students were asked “What is your religion?”. They could chose among Christian, Islamic, other and none and in a second step specify the denomination of Christianity or Islam. In Slovakia, there was just one question with the three major Christian denominations, other religions and none as response options.

#### Perceived stress

This concept was measured with the four-item version of the Cohen’s perceived stress scale (PSS). PSS-4 is an economical and simple psychological instrument that measures the degree to which situations in one’s life over the past month are appraised as stressful [45]. The questions are of a general nature and items are designed to detect how unpredictable, uncontrollable, and overloaded respondents find their lives, e.g. “How often have you felt that you were unable to control the important things in your life?”; and, “How often have you felt confident about your ability to handle your personal problems?”. Students responded on a five-point scale (0 = “never”, 1 = “almost never”, 2 = “sometimes”, 3 = “fairly often”, 4 = “very often”). Items were recoded so that higher scores indicated more perceived stress. Cronbach’s alpha coefficients were 0.74 (Germany), 0.75 (Poland), 0.67 (Bulgaria), 0.50 (UK) and 0.54 (Slovakia). The PSS score was obtained by summing up answers to individual questions.

#### Depressive symptoms

These were measured using a modified version of the Beck Depression Inventory (M-BDI) [46,47]. Students were asked to describe how often they experienced 20 depressive feelings during the past few days with 6-point scale responses (from 0=”never” to 5 = “almost always”). The M-BDI score is obtained by summing up answers to individual questions. The authors of the M-BDI have demonstrated its construct validity and measurement equivalence as compared to the original BDI, and provided a cut-off score (at 35 points) for screening for clinically relevant depressive symptoms. The scale showed good to acceptable levels of reliability in all participating countries. Cronbach’s alpha coefficients were 0.90 (Germany), 0.92 (Poland), 0.87 (Bulgaria), 0.92 (UK) and 0.89 (Slovakia).

#### Religiosity (personal importance of religious faith)

From the many options available to measure formal and informal religiosity [48], we prioritised the assessment of the personal importance of religious faith as a measure of religiosity. Participants were asked about the extent that they agreed/disagreed with the following statement: "My religion is very important for my life”. Response options (1 = “strongly disagree”, 2 = “somewhat disagree”, 3 = “neither agree nor disagree”, 4 = “somewhat agree” and 5 = “strongly agree”) were subsequently dichotomised into two categories: ‘low importance of religious faith’ (values 1, 2 and 3) and ‘high importance of religious faith’ (values 4 and 5).

### Statistical analysis

Listwise deletion was performed for handling missing data. Descriptive analysis of alcohol consumption, problem drinking and other studied variables was undertaken separately for each country. In the next step, the association between stress or depressive symptoms on the one side and frequency of drinking or problem drinking on the other was analysed in four separate multivariable logistic regression models. All models included sex, country, perceived sufficiency of income and personal religiosity. Additionally, all two-way interactions and three-way interactions were assessed. Only significant interactions were reported and included in final tables. All analyses were conducted using SPSS 16, findings were reported as odds ratios (OR) with 95% confidence intervals (CI). Significance level was set at p <0.05.

## Results

### Characteristics of the sample

The main characteristics of the study population are presented in Table 1. Across the five countries, the majority of participants were females, with relatively higher proportions of female students in the Slavic countries. Slovakian students were the youngest, followed by students from Bulgaria, Poland, the UK, and Germany. Because of the relative small age variability within the countries and a higher variability between the countries, no adjustment for age was performed in the subsequent analyses. About 59.3% of the UK freshmen drank alcohol once a week or more often, followed by Bulgaria (33.6%), Germany (26.9%), Slovakia (15.1%) and Poland (12.2%). Problem drinking (two or more positive responses in CAGE) was found in 22.1% of UK students, followed by Slovakia (21.2%), Germany (17.0%), Bulgaria (13.6%) and Poland (11.8%). Importance of religious faith was highest among Polish participants followed by Slovakian, UK, Bulgarian and German participants. As expected, there was a substantial variation in students’ religions by country, with 21% of students stating no religion in Slovakia, 15.7% in Bulgaria, 12.1% in Germany and only 1.0% in Poland (data for the UK were not available). Among those who stated religion, most common was Christianity, but there were substantial differences in denominations, with Catholics being most common in Poland and Slovakia, Orthodox Christians in Bulgaria and Protestants in Germany.

**Table 1:** Description of the sample across participating sites

	Bielefeld, Germany N = 650	Lublin, Poland N = 554	Sofia, Bulgaria N = 684	Gloucestershire, UK N = 300	Kosice, Slovakia N = 315
Categorical variables					
Female (%)	57.8	71.1	68.4	60.3	70.5
High frequency of drinking (%)^1^	26.9	12.2	33.6	59.3	15.1
Problem drinking (%)^2^	17.0	11.8	13.6	22.1	21.2
High importance of faith (religiosity) (%)^3^	16.2	75.5	15.4	20.7	54.8
Religion (%)					
None	12.1	1.0	15.7	― ^8^	21.0
Christian					
Catholic	25.7	83.8	0.8	― ^8^	67.2
Protestant	35.7	0.0	1.3	― ^8^	5.0
Orthodox	1.0	0.2	35.5	― ^8^	1.8
Unspecified^4^	17.7	11.2	41.0	― ^8^	―
Other (non-Christian)	2.6	0.5	2.4	― ^8^	2.7
Missing	5.2	3.4	3.2	― ^8^	2.2
Score variables					
Subjective sufficiency of income [mean (SD)]^5^	2.34 (0.86)	2.46 (0.86)	2.60 (0.80)	2.54 (0.86)	2.29 (0.90)
Depressive symptoms [mean (SD)]^5^	26.84 (15.23)	31.84 (17.11)	32.60 (14.98)	23.03 (15.21)	28.70 (15.42)
Cohen’s perceived stress score [mean (SD)]^7^	8.05 (1.69)	8.04 (1.85)	8.24 (2.07)	6.76 (2.31)	9.00 (2.09)

^1^ Drinking a few times each week and more.

^2^ ≥2 positive answers in CAGE.

^3^ “My religion is very important for my life” (1 = “strongly disagree”, 5 = “strongly agree”. High importance of religious faith (religiosity) = values 4 and 5.

^4^ Students in Germany, Poland and Bulgaria could first choose the religion and in an additional question specify the denomination. The unspecified group includes those who chose Christianity, but did not specify the Christian denomination. Probably, these students also belong to the denomination of the local majority. A similar two-step description of religion was asked for Islam, but due to low numbers, the results are not reported separately. In Slovakia there was a single question which asked about the three major Christian denominations, other religions and none.

^5^ “How sufficient is your income?” (1 = “totally sufficient”, 4 = “not sufficient at all”). Larger numbers indicate that income was more often perceived as insufficient.

^6^ Sum of 20 items scored from 0 to 5. Maximum of 100 indicates strongest depressive symptoms.

^7^ Sum of four items scored from 0 to 4. Maximum of 16 indicates highest perceived stress.

^8^ Due to a coding error for the Anglican Church, the UK data on religion could not be analysed.

Subjective perception of sufficiency of income was similar across the countries, with highest scores in the UK and Bulgaria and lowest in Slovakia and Germany. Mean PSS scores were highest in Slovakia followed by Bulgaria, Poland, Germany and the UK. The M-BDI scores were highest for the Bulgarians followed by Poles, Slovaks, Germans and finally the UK students.

### Association between perceived stress and high frequency of alcohol consumption and problem drinking

Perceived stress was not associated with high frequency of drinking, but was associated with problem drinking after adjusting for sex, country, perceived sufficiency of income and importance of religious faith (Table 2). There was a significant interaction between sex and importance of religious faith: For both, the high frequency of drinking and problem drinking, females had a lower risk, additionally, the protective effects of high importance of religious faith were stronger among females than males. The association between perceived stress and problem drinking differed across the studied countries, with less pronounced effects in Germany and Slovakia and more pronounced effects in the UK. The interaction between the importance of religious faith and perceived stress was not significant; hence, there was no evidence that importance of religious faith modifies the relationships between perceived stress and high frequency of alcohol consumption or problem drinking.

**Table 2:** **Associations between perceived stress and high frequency of alcohol consumption and with problem drinking in university freshmen from five European countries**^**a**^

	**High frequency of drinking**				**Problem drinking (CAGE > 2)**			
	**Wald**^**+**^	**df**	**p**	**OR (95%CI)**	**Wald**^**+**^	**df**	**p**	**OR (95%CI)**
Perceived stress (per unit)^1^	0.06	1	0.809	1.01(0.96-1.07)	6.28	1	0.012	1.16(1.03-1.30)
Sex								
Male				1				1
Female	56.45	1	<0.001	0.19(0.12-0.29)	39.12	1	<0.001	0.24(0.15-0.37)
Country	120.56	4	<0.001		22.39	4	<0.001	
Bulgaria				1				1
Germany	4.32	1	0.038	0.76(0.59-0.99)	7.96	1	0.005	1.67(1.04-2.70)
Poland	26.45	1	<0.001	0.37(0.25-0.54)	0.34	1	0.563	2.25.(.17-28.99)
UK	53.25	1	<0.001	3.55(2.53-4.98)	0.05	1	0.824	0.98(0.08-11.59)
Slovakia	8.70	1	0.003	0.55(0.37-0.82)	16.19	1	<0.001	94.88(9.10-989.28)
Perceived income sufficiency (per one point change)^2^	4.27	1	0.039	1.02(1.00-1.03)	1.64	1	0.200	1.01(0.99-1.02)
Importance of religious faith (religiosity)^3^								
Low				1				1
High	2.72	1	0.099	0.72(0.49-1.05)	2.32	1	0.128	0.73(0.49-1.09)
Interactions								
Perceived stress * Bulgaria				N.S.				1
Perceived stress * Germany				N.S.	6.58	1	0.10	0.79(0.66-0.95)
Perceived stress * Poland				N.S.	0.49	1	0.482	0.93(0.76-1.14)
Perceived stress * UK				N.S.	0.24	1	0.625	1.05(0.85-1.29)
Perceived stress * Slovakia				N.S.	10.54	1	0.001	0.74(0.61-0.89)
Sex * importance of religious faith	12.65	1	<0.001	2.48(1.52-4.07)	5.15	1	0.023	1.86(1.08-3.17)

^a^ Findings from logistic regression models predicting high frequency of drinking and problem drinking, adjusted for all variables in the Table.

^**+**^ Wald chi-square test.

N.S. – not significant.

^1^ Sum of four items scored from 0 to 4. Maximum of 16 indicates highest perceived stress.

^2^ “How sufficient is your income?” (1 = “totally sufficient” to 4 = “not sufficient at all”).

^3^ “My religion is very important for my life” (1 = “strongly disagree” to 5 = “strongly agree”. High importance of religious faith (religiosity) = values 4 and 5.

### Association between depressive symptoms and high frequency of alcohol consumption and problem drinking

Depressive symptoms were not associated with high frequency of drinking, but were associated with problem drinking after adjusting for sex, country, perceived sufficiency of income and importance of religious faith (Table 3). Similarly to perceived stress, there was a significant interaction between sex and importance of religious faith for both, the high frequency of drinking and problem drinking. The association between depressive symptoms and both alcohol-related variables did not differ by country or sex.

**Table 3:** **Association between depressive symptoms and a high frequency of alcohol consumption and with problem drinking in university freshmen from five European countries**^**a**^

	**High frequency of drinking**				**Problem drinking (CAGE > 2)**			
	**Wald**^**+**^	**df**	**p**	**OR (95%CI)**	**Wald**^**+**^	**df**	**p**	**OR (95%CI)**
Depressive symptoms (per 10 points)^1^	0.48	1	0.655	1.03 (0.95-1.11)	34.34	1	<0.001	1.26 (1.17-1.37)
Sex								
Male				1				1
Female	47.23	1	<0.001	0.21 (0.13-0.33)	40.88	1	<0.001	0.23 (0.15-0.37)
Country	116.56	4	<0.001		21.78	4	<0.001	
Bulgaria				1				1
Germany	4.44	1	0.045	0.76 (0.58-0.99)	5.89	1	0.017	1.52 (1.08-2.14)
Poland	19.93	1	<0.001	0.39 (0.26-0.59)	0.39	1	0.684	1.10 (0.70-1.71)
UK	51.59	1	<0.001	3.32 (2.35-4.70)	10.59	1	0.008	1.83 (1.17-2.84)
Slovakia	12.48	1	0.003	0.54 (0.36-0.81)	14.71	1	<0.001	2.35 (1.56-3.55)
Perceived income sufficiency (per one point change)^2^	1.61	1	0.043	1.02 (1.01-1.03)	0.48	1	0.178	1.01 (0.99-1.02)
Importance of religious faith (religiosity)^3^								
Low				1				1
High	1.36	1	0.185	0.75 (0.50-1.12)	1.65	1	0.147	0.73 (0.48-1.12)
Interactions								
Sex*religious faith	9.30	1	0.005	2.11 (1.27-3.52)	6.07	1	0.024	1.88 (1.09-3.24)

^a^ Findings from logistic regression models predicting high frequency of drinking and problem drinking, adjusted for all variables in the table.

^**+**^ Wald chi-square test.

^1^ Sum of 20 items scored from 0 to 5. Maximum of 100 indicates strongest depressive symptoms.

^2^ “How sufficient is your income?” (1 = “totally sufficient”, 4 = “not sufficient at all”. Larger numbers indicate that income was more often perceived as insufficient.

^3^ “My religion is very important for my life.” (1 = “strongly disagree”, 5 = “strongly agree”. High importance of religious faith = values 4 and 5.

## Discussion

This study analysed the relationship between perceived stress and depressive symptoms and alcohol use and the modifying role of religious faith. We used two variables describing distinct patterns of alcohol use. Alcohol-related health and social problems tend to increase as the frequency of alcohol consumption rises [49], hence the first variable that the current study employed is the frequency of drinking, which is a general indicator that does not assess the quantity of alcohol consumed. The second variable is problem drinking, which focuses more on long-term drinking habits and thus represents more serious drinking patterns that have potentially more negative consequences [50,51]. Such use of two distinct variables produces a more detailed picture of alcohol consumption across countries, and a more holistic understanding of its relationships to mental health indicators and religiosity.

Overall, the study found more high frequency drinking in Western (UK and Germany) and Southern (Bulgaria) European countries than in Central European countries (Slovakia and Poland). Problem drinking was most common in the UK and in Slovakia and least common in Bulgaria and Poland. Our results agree with WHO statistics of per-capita consumption of alcohol in these countries [52].

In relation to perceived stress, several studies of stress and substance use among college student populations provide evidence that stress motivates alcohol consumption [53-56]. Students experiencing higher levels of stress tend to use alcohol and other substances at higher levels and have a higher number of substance-related problems [54,55]. We confirm this association among university students from five European countries for problem drinking. At the same time, there was no such association for frequency of drinking, which can be explained by the fact that the frequency of drinking is either not an indicator of alcohol problems yet, or that there is a strong influence of local cultures of drinking with regular but not problematic drinking (e.g. regular consumption of wine with meals in Bulgaria as part of a Southern European culture). The association between perceived stress and problem drinking was weaker among students in Germany and Slovakia. It could be that students from these countries have more/other alternative coping mechanisms and thus when encountering stress are less likely to develop problem drinking. Consistent with past studies of college students [3], our findings confirm the positive association between depressive symptoms and problem drinking among university students. Problem drinking can be a consequence of depressive symptoms [21], but the relationship works probably in both ways, so that drinking may lead to depressive symptoms [57].

Problem drinking among college students may be a means of coping with perceived stress and may also vary in relation to coping abilities e.g. religious coping. Results from current study are partly consistent with previous research demonstrating that religious faith was inversely related to the quantity and frequency of alcohol use and problem drinking in samples of college students [28,34,58]. In line with a previous study [59] we also found gender differences in religiosity with respect to the high frequency of drinking and with problem drinking. Females with a high level of religiosity were less likely to be involved in high frequency drinking and problem drinking compared to males. This relationship was consistent across countries. Females generally report higher levels of religiosity than males [60,61], which could be related to different socialization, expected roles, and coping strategies relative to males. Religion may be more important for females and consequently more likely to influence their risk behaviours [62]. On the other side, religiosity did not modify the association between perceived stress or depressive symptoms and alcohol consumption or problem drinking. Given the different religious traditions with majority status in each country, a further interesting finding of the current study is that the effects of religiosity did not differ by country. This supported the notion that religiosity was more important with respect to alcohol consumption than the actual faith/denomination.

### Limitations

There are several limitations to our study. Despite relatively low Cronbach’s alphas off PSS4 for the UK and Slovakia, we decided to keep these countries in the analyses. The alpha coefficient increases with the instrument's length [63]; and it has been argued in previous studies that a reliability coefficient as low as 0.5 should not seriously attenuate validity [64]. Still, the question remains why PSS4 performed worse in the UK and in Slovakia. The results related to prevalence of problem drinking have to be treated with caution as the CAGE test appears not to be an ideal measure of problem drinking in university students [65,66]. However, the focus of our analysis was not on quantifying problem drinking, but on comparing those with higher and lower scores regarding problem drinking. The relative relationship can remain meaningful even if absolute scores require a different cut-off.

We used a single measure of religiosity. Religiosity is a multi-dimensional construct (i.e. spirituality, beliefs, practices, religious affiliation and attendance) [48], and restricting it only to the dimension of personal importance of faith, which can be viewed as a dimension of spirituality, might have affected the observed relationship between religiosity and use of alcohol [67]. Similarly, given the self-reported measures of drinking, some underreporting, e.g. for problem drinking which is socially undesirable, might have occurred especially among the religious groups of participants. Another limitation is the use of alcohol consumption frequency alone, without consideration of the amount consumed on each occasion. The relationship between depressive symptoms and alcohol use may be different for someone who has one drink every day compared with someone who drinks heavily every day.

Regarding the time window of three months for the question on the frequency of alcohol consumption, recall bias also needs to be considered. In addition, each of the applied measures included a different recall period (e.g., past 3 months for frequency of alcohol consumption, lifetime for problem drinking, past few days for depressive symptoms, and past month for perceived stress). This further complicates the already difficult task of interpreting the findings from cross-sectional data in terms of temporality. For example, although problem drinking was regressed on depressive symptoms in the logistic regressions, the experience of alcohol problems may have occurred earlier in a student’s life (e.g. in adolescence) than the recent experience of depressive symptoms. We also did not address the issue of other potential variables associated with alcohol consumption and mental health. For example, it has been suggested in previous studies that when controlling for the effects of anger, depressed mood has little, or no independent effects on alcohol use [68]. Unfortunately, we did not have a measure of emotions such as anger in our questionnaire. Another concern can be the use of just a subjective measure of income sufficiency, instead of an objective amount of income. In one previous publication, we compared the subjective income sufficiency with reported income and concluded that for international comparisons with respect to mental health aspects the latter can be more useful [39].

The sample comprised first-year students. Thus, any generalisations of the findings for all students should be undertaken cautiously. We included only one university per country (except for Slovakia), so the observed differences might be specific site characteristics rather than true differences between countries. Future research should address these limitations.

## Conclusions

We confirmed the previously proposed association between perceived stress and depressive symptoms and problem drinking in a culturally different sample of European students from five countries. At the same time, we demonstrated that the association does not exist for the frequency of alcohol consumption. We also showed that religiosity plays a role within this association, but the interaction effect is restricted to females.

These findings should be taken into account when developing prevention programs for problem drinking among students. The policy recommendation for addressing problem drinking should include improvement of mental health and development of coping mechanisms. We studied the effects of religiosity and found out that its role was limited to females, but other coping strategies can be more universal. Many students can ‘feel down’ sometimes. For adolescents and young adults, maladaptive coping mechanisms e.g. drug or alcohol use are common when dealing with social and emotional problems. Such coping strategies are ineffective and provide only immediate relief from stressful situations, and may even exacerbate the problems that the person is currently experiencing. Talking openly, having appropriate social support and adequate coping skills can help prevent the transformation of periods of sadness to more severe depression.

Prevention programs should directly target specific risk factors (e.g. perceived stress, depressive symptoms) that impact on adolescent well-being and focus on implementation programs that teach adaptive coping responses and problem solving skills so that they can effectively handle problems and stressors that typically characterise university students’ lives.

## Competing interest

The authors declare no conflict of interest.

## Authors’ contributions

RS developed the research question, performed the statistical analyses and interpretation of the data, and drafted the manuscript. WEA wrote considerable parts of the manuscript and together with CS and OO commented extensively on the manuscript. RTM developed the study design, supervised the analysis, contributed to the interpretation and writing. All the authors read and approved the final version of the manuscript.
